# Immunogenicity of Non-Mutated Ovarian Cancer-Specific Antigens

**DOI:** 10.3390/curroncol31060236

**Published:** 2024-05-30

**Authors:** Leslie Hesnard, Catherine Thériault, Maxime Cahuzac, Chantal Durette, Krystel Vincent, Marie-Pierre Hardy, Joël Lanoix, Gabriel Ouellet Lavallée, Juliette Humeau, Pierre Thibault, Claude Perreault

**Affiliations:** 1 Institute for Research in Immunology and Cancer (IRIC), University of Montreal, Montreal, QC H3T 1J4, Canada; leslie.hesnard@umontreal.ca (L.H.); catherine.theriault.4@umontreal.ca (C.T.); maxime.cahuzac@umontreal.ca (M.C.); chantal.durette@umontreal.ca (C.D.); krystel.vincent@umontreal.ca (K.V.); marie-pierre.hardy@umontreal.ca (M.-P.H.); joel.lanoix@umontreal.ca (J.L.); gabriel.ouellet.lavallee@umontreal.ca (G.O.L.); juliette.f.humeau@gmail.com (J.H.); pierre.thibault@umontreal.ca (P.T.); 2 Department of Chemistry, University of Montreal, Montreal, QC H2V 0B3, Canada; 3 Department of Medicine, University of Montreal, Montreal, QC H3C 3J7, Canada

**Keywords:** tumor-specific antigen, vaccine, mass spectrometry, antigen presentation, immunogenicity, dendritic cells

## Abstract

Epithelial ovarian cancer (EOC) has not significantly benefited from advances in immunotherapy, mainly because of the lack of well-defined actionable antigen targets. Using proteogenomic analyses of primary EOC tumors, we previously identified 91 aberrantly expressed tumor-specific antigens (TSAs) originating from unmutated genomic sequences. Most of these TSAs derive from non-exonic regions, and their expression results from cancer-specific epigenetic changes. The present study aimed to evaluate the immunogenicity of 48 TSAs selected according to two criteria: presentation by highly prevalent HLA allotypes and expression in a significant fraction of EOC tumors. Using targeted mass spectrometry analyses, we found that pulsing with synthetic TSA peptides leads to a high-level presentation on dendritic cells. TSA abundance correlated with the predicted binding affinity to the HLA allotype. We stimulated naïve CD8 T cells from healthy blood donors with TSA-pulsed dendritic cells and assessed their expansion with two assays: MHC-peptide tetramer staining and TCR Vβ CDR3 sequencing. We report that these TSAs can expand sizeable populations of CD8 T cells and, therefore, represent attractive targets for EOC immunotherapy.

## 1. Introduction

Epithelial ovarian cancer (EOC) is the leading cause of gynecological cancer death. High-grade serous ovarian carcinoma represents 70% of EOC and accounts for 90% of advanced-stage disease and mortality [1,2]. The late diagnosis is one of the main factors contributing to a low 5-year survival rate recently estimated at 50% [3]. EOC is considered a tumor type with potential immunoreactivity due to the well-documented positive correlation between the presence of tumor-infiltrating lymphocytes and better clinical outcomes [4,5,6,7,8]. However, immune checkpoint inhibitors have demonstrated limited efficacy in EOC [7,9], and the lack of well-defined actionable antigens has hampered the development of therapeutic vaccines [7,10].

The dominant paradigm holds that anti-tumor immune responses enhanced by immune checkpoint therapies are primarily directed toward tumor-specific antigens (TSAs) [11,12,13]. There are two types of TSAs: mutated TSAs (mTSAs) and aberrantly expressed TSAs (aeTSAs). mTSAs derive from mutated DNA sequences, typically located in exons of protein-coding genes. In contrast, aeTSAs result from the translation of any open reading frames not expressed in normal adult somatic cells. Their presence in cancer cells arises from cancer-specific epigenetic changes and splicing aberrations [13,14,15,16,17]. So far, cancer vaccine research has given considerable attention to mTSAs. However, mass spectrometry (MS) analyses failed to detect most predicted mTSAs on the surface of tumor cells [18,19,20,21]. To identify TSAs, we developed a proteogenomic approach that integrates transcriptomic and MS analyses. MS validation represents the most solid evidence that TSAs are genuinely presented by cancer cells [19,22,23]. Using this method, we reported that, in EOC and other tumor types, most TSAs belong to the aeTSA category [14,17,24,25], making their evaluation of particular interest in cancer vaccine development.

Optimal targets for cancer vaccine development should possess two main features [26,27]. First, they should be cancer-specific. Indeed, TSAs are expected to be immunogenic because cognate T cells escape central immune tolerance. Second, optimal targets should be shared by a significant proportion of EOCs, thereby facilitating the development of off-the-shelf cancer vaccines and TCR-based biologics. Most EOC aeTSAs are shared by a substantial proportion of tumors [24]. In the present study, we aimed to evaluate the therapeutic potential of these TSAs against EOCs by assessing their capacity to induce antigen (ag)-specific T-cell responses. To this end, we stimulated naïve CD8 T cells with dendritic cells pulsed with synthetic TSA peptides. We then assessed TSA presentation by MS analyses and estimated T-cell responses using TCR Vβ CDR3 sequencing and tetramer staining.

## 2. Materials and Methods

### 2.1. Selection of the Most Prevalent TSAs

Starting from the list of 91 TSAs identified in ovarian cancer [24], we prioritized 48 peptides predicted to be highly shared across patients based on two criteria: (i) expression by a sizeable proportion of EOCs from The Cancer Genome Atlas (TCGA) dataset, and (ii) presented by a high-frequency HLA-A or HLA-B allotype.

#### 2.1.1. Selection of Highly Prevalent TSAs

The predicted binding affinity (in nM) and rank (%) of each peptide for the relevant HLA allotype were obtained from the NetMHCpan4.1 algorithm [28] (the lower the value, the higher the affinity). The percentile rank threshold for predicted strong binders is inferior or equal to 0.5%. The predicted half-life stability for peptide–HLA complexes (in hours) was determined from the NetMHCstabpan1.0 database [29] (Table S1).

For each TSA, the HLA molecule used in this study was assigned from MS identifications [24], with one exception: IIHSSSLLL (p10), initially eluted from HLA-C*07:01, was found in the present study to bind HLA-A*02:01 based on NetMHCpan4.1 eluted ligand likelihood prediction (Table S1). In total, nine HLA allotypes were included in the present study: HLA-A*01:01, -A*02:01, -A*03:01, -A*11:01, -A*29:02, -B*07:02, -B*08:01, -B*15:01, and -B*44:03 (Table S2). Their prevalence in four populations (Caucasian, African, Hispanic, and Asian/Pacific Islanders) was calculated using published allele frequency data [30] and the following mathematical formula: prevalence = [F^2^ + 2 (F (1 − F))] × 100 where F corresponds to allele frequency.

#### 2.1.2. Evaluation of Cancer Specificity and Sharing of TSA Source Transcripts

**Figure 1:**
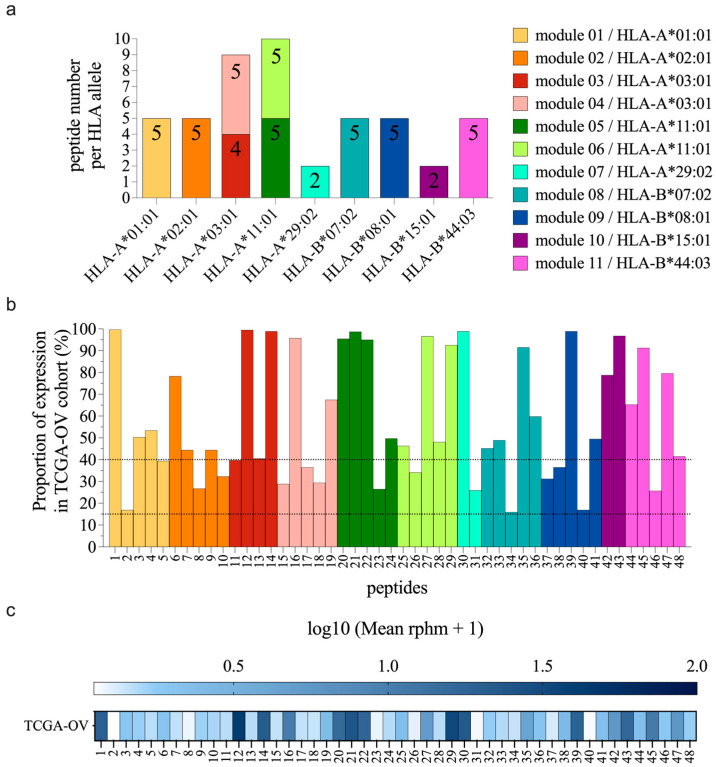
48 TSAs presented by nine common HLA allotypes and highly shared by ovarian tumors. (**a**) Number of peptides included per HLA allotype and repartition in 11 vaccine modules. Bars indicate the number of peptides in each module. (**b**) Proportions of samples from the TCGA ovarian cohort expressing the peptide coding sequences (rphm > 0) for each TSA. The dotted lines represent sharing frequencies of 15% and 40%. (**c**) Heat map representing peptide coding sequences expression (log(rphm + 1)) in the TCGA ovarian cohort (mean from 377 samples).

Using BamQuery [31] in manual mode, we quantified the expression of TSA-coding transcripts in ovarian cancer samples (TCGA, n = 377; https://www.cancer.gov/ccg/research/genome-sequencing/tcga, accessed on 8 March 2017) and normal tissues [(Genotype-Tissue Expression cohort (GTEx) [32] and medullary thymic epithelial cells (mTECs) samples [14,33,34]; Gene Expression Omnibus (GEO) GSE127825 and GSE127826)]. We estimated the proportion of the TCGA ovarian samples with a TSA-coding sequence expression higher than two times the normal 95th percentile value. The normal 95th percentile value corresponds to the 95th percentile expression value calculated from all normal tissue samples used in the study (mTECs (n = 11) + GTEx (except testis) (n = 2439)). The proportion of TCGA ovarian samples expressing TSA-coding sequence (rphm > 0) was evaluated and represented in Figure 1b and Table S1. The expression of the TSA coding sequences (log(rphm + 1)) in the TCGA ovarian cohort (mean from 377 samples) was also calculated to be represented in the heat map (Figure 1c and Table S1).

TSAs were then divided into modules based on their allelic presentation, with a maximum of 5 TSAs per module. For HLA allotypes with more than five selected TSAs (HLA-A*03:01 and -A*11:01), TSAs were split into two groups according to their predicted binding affinity to minimize the binding competition between peptides (see Figure 1a and Table S1). The key features of selected peptides are compiled in Table S1.

### 2.2. Peripheral Blood Mononuclear Cell Isolation

Leukapheresis products from healthy donors expressing one or several HLA alleles of interest were purchased from BioIVT (Westbury, NY, USA) or Miltenyi Biotec (San Diego, CA, USA). Details on HLA alleles from all healthy donors used in this study are compiled in Table S3. Peripheral Blood Mononuclear Cells (PBMCs) were then isolated using Ficoll Gradient (Cytiva, Vancouver, BC, Canada, #cat.17144003) or the MultiMACS Cell24 Separator Plus (Miltenyi Biotec, #cat. 130-098-637) in conjugation with the Straight From Leukopak PBMC Isolation Kit (Miltenyi Biotec, #cat. 130-123-456) according to the manufacturer’s protocol. Isolated PBMCs were frozen in liquid nitrogen in FBS-10% DMSO until use.

### 2.3. Generation of Monocyte-Derived Dendritic Cells

Monocyte-derived dendritic cells (moDCs) were generated from frozen PBMCs, as previously described [35]. Briefly, moDCs were obtained from the CD14+ enriched PBMC fraction using the CD14+ microbeads from Miltenyi Biotec (#cat. 130-050-201) (day-5) as per the manufacturer’s protocol. Monocytes were then cultured for five days in complete X-vivo 15 medium (Lonza Bioscience, Montreal, QC, Canada, #cat. 04-418Q): 5% human serum (Sigma-Aldrich, St. Louis, MO, USA, #cat. H4522-100 mL), 1 mM Sodium pyruvate (Gibco, Thermo Fisher scientific, Agawam, MA, USA, #cat. 11360070), 1% penicillin-streptomycin (Gibco) supplemented with IL-4 (50 UI/mL, Peprotech, Cranbury, NJ, USA, #cat. 200-04-250UG) and GM-CSF (800 UI/mL, Peprotech, #cat. 300-03-1MG). After 72 h (on day-2), complete X-vivo 15 medium was added to the plates supplemented with cytokines to reach IL-4 and GM-CSF final concentrations (50 IU/mL and 800 IU/mL, respectively). After five days of culture (day-1), moDCs were matured overnight with IFN-γ (100 IU/mL, Peprotech, #cat. 300-02-1MG) and LPS (10 ng/mL, Sigma Aldrich, #cat. L4524-5MG). On collection day, mature moDCs were put on ice for 20 min and then gently removed from the plasticware using a cell scraper (VWR, Ville Mont-Royal, QC, Canada, #cat. 10062-908). Harvested moDCs were washed with cold PBS. For moDCs added after ten days of coculture, moDC generation was initiated four days before the second round of stimulation. Some media was added after 48 h, and maturation was launched the day before their addition to the coculture.

### 2.4. Peptide Pulsing and Electroporation of moDCs

For peptide pulsing, moDCs were incubated for two hours with complete X-vivo 15 medium supplemented (pulsed) or not (unpulsed) with synthetic peptides (in pools or individually) purchased from GL Biochem (10 µg/mL each). After two washes, moDCs were used as APCs in naïve CD8 T cells-moDCs coculture or as dry pellets for MS experiments. Of note, to take into account the deaminated-N1 modification of p40 (DILGKSLTL) detected in the initial MS analysis [24], in vitro experiments were performed using the synthetic peptide NILGKSLTL (Table S1).

For electroporation experiments, pT7pA100 plasmids with a sequence coding for five peptides of interest (modules 1, 2, or 6) were designed and purchased from GenScript. We followed the protocol described by Ali and colleagues [35] with a few modifications. Briefly, plasmids were digested with MfeI-HF enzyme (New Englands Biolab, Ipswich, MA, USA, #cat. R3589S) for four hours at 37 °C and purified with Qiagen PCR Purif Kit (Qiagen, Germantown, MD, USA, #28104). Transcription was performed using the Ribomax Large Scale RNA Production Systems—T7 (Promega, Madison, WI, USA, #cat. P1300) following the manufacturer’s instructions. RNA was capped with Ribo m7G Cap Analog 25 A254 Units (Promega, #cat. P1712) and purified using the Monarch^®^ RNA Cleanup Kit (New Englands Biolab, #cat. T2050L). RNA was stocked at −80 °C until use. A total of 5 million moDCs were electroporated with 100 µg of purified RNA using the BTX ECM830 electroporation system (BTX Harvard Apparatus, Holliston, MA, USA) in 4.0 mm electrogap cuvettes (VWR, #cat. CA58017-900). Electroporation was performed using the following parameters: 500 volts and one pulse of 2 milliseconds. After electroporation, moDCs were immediately transferred in 6-well plates (Sarstedt, Nümbrecht, Germany, #cat. 83.3920300) with fresh media (complete X-vivo 15 medium, see Section 2.3) and put back in the incubator at 37 °C and 5% CO_2_. Six hours later, moDCs were used as APCs in naïve CD8 T cells-moDC cocultures.

### 2.5. MS Experiments

#### 2.5.1. Cell Sample Preparation

Ten million mature moDCs per condition were pulsed with a mix of peptides corresponding to each module at a concentration of 10 µg/mL each. Unpulsed mature moDCs (negative control) and mature moDCs pulsed with a pool of control peptides mainly from viruses (module 12) were also included. Details about control peptides (module 12) are compiled in Table S4. After peptide pulsing for two hours at 37 °C, moDCs were harvested, washed twice in cold PBS, and centrifuged twice at 300× *g* for ten minutes. Dried pellets were kept at −80 °C until immunoprecipitation of MHC-I-associated peptides. Each TSA module was done in 3 biological replicates (3 different donors) and at two different time points (t = 0 h and 24 h after pulsing). The control module (module 12) was tested in all donors included in these experiments (see Table S3 for details) at time zero after peptide pulsing.

#### 2.5.2. Immunoprecipitation of HLA Class I Molecules

The W6/32 antibodies (BioXcell, Lebanon, NH, USA) were coupled to CNBR-activated Sepharose 4B beads (Cytivia, Vancouver, BC, Canada) as described [36,37], and the beads were stored at 4 °C in PBS pH 7.2 and 0.02% NaN_3_ until use. Cell pellets were resuspended with 0.7 mL of PBS and then solubilized by adding 1 mL of ice-cold 1% *w*/*v* CHAPS (Sigma, Oakville, ON, Canada) buffer supplemented with a Protease inhibitor cocktail (Sigma). After incubation for 1 h with tumbling at 4 °C, tissue samples were spun at 16,600× *g* for 20 min at 4 °C. Supernatants were transferred into new tubes containing 1 mg of W6/32 antibody covalently-cross-linked to Sepharose beads and incubated with tumbling for 3 h at 4 °C. The samples were transferred into poly prep chromatography columns (Biorad, Mississauga, ON, Canada), and the liquid mixture was eluted by gravity. Sepharose beads were washed twice with PBS, then twice with 0.1× PBS, and finally twice with water. MHC-I complexes were eluted from sepharose beads by acidic treatment using 1% trifluoroacetic acid (TFA, Sigma-Aldrich). Filtrates containing peptides were separated from MHC-I subunits (heavy chain alpha of HLA molecules and β-2 microglobulin) using homemade stage tips packed with two 1 mm diameter octadecyl (C-18) solid-phase extraction disks (EMPORE, Bioanalytical Technology 3M Company, St-Paul, MN, USA). Stage tips were pre-washed first with methanol, then with 80% acetonitrile (ACN, Fisher Chemicals, Ottawa, ON, Canada) in 0.1% TFA, followed by 0.1% TFA and finally with 1% TFA. Samples were loaded onto the stage tips, and the peptides were retained on the stage tips while the alpha chains from HLA molecules and β-2 microglobulin were found in the flow-through. Stage tips were washed with 1% TFA and then with 0.1% TFA, and peptides were eluted with 30% ACN in 0.1% TFA. The peptides were dried using vacuum centrifugation and stored at −20 °C until MS analysis.

#### 2.5.3. Mass Spectrometry Analyses

Synthetic peptides (GL Biochem, Shanghai, China) were dissolved in DMSO at 1 nmol/μL and diluted at 0.25 pmol/μL in 4% formic acid. From these stock solutions, peptides were combined, and serial dilutions were performed to obtain decreasing concentrations for the calibration curve. Targeted MS/MS was performed on an Exploris 480 interface with an Easy-nLC 1200 (Thermo Scientific, Waltham, MA, USA). Dried peptide extracts were resuspended in 4% formic acid and were loaded on a C4 precolumn (Optimize Technologies, Oregon City, OR, USA) and separated on a 20 cm × 150 μm homemade Jupiter C18 (Phenomenex, Torrance, CA, USA) 3 μm, 300 Å column. Elution was performed using a 56 min linear gradient of 7% to 30% aqueous acetonitrile, ACN (0.1% formic acid) at a flow rate of 600 nL/min. Survey scan resolution, automatic gain control, and injection time were respectively set at 120 K, 1 × 10^6^, and Auto over a scan range of 300−650 *m*/*z*. Targeted MS scans were run with an inclusion list of the peptide module being analyzed, a resolution of 45 K, a normalized AGC target of 100%, and an HCD normalized collision energy of 34. Samples of the calibration curve were analyzed using the same method as samples, except the inclusion list contained all peptides of the 12 modules, and a retention time window was used.

#### 2.5.4. Bioinformatic Analysis

Peptides were identified with PEAKS X Pro (Bioinformatics Solutions, Waterloo, ON, Canada) with a tolerance of 10 ppm and 0.01 Da for precursors and fragments, respectively, against a tailored database of 48-peptide sequences to build a library for Skyline. All data were analyzed with Skyline. Peptide detection and quantitation were further performed with Skyline 22.2.0.312 using high selectivity extraction. Based on calibration curves, a limit of quantification was calculated for each peptide. Peptides detected below this limit were listed as detected but not precisely quantified.

### 2.6. Immunogenicity Testing

#### 2.6.1. Coculture of Naïve CD8 T Cells with moDCs

Thawed PBMCs from healthy donors were naïve CD8 T cell-enriched, as previously described [35], using either (i) the Human CD8 T cell isolation kit from Miltenyi Biotec (#cat. 130-096-495) combined with CD45RO (Miltenyi Biotec, #cat. 130-046-001) and CD57 microbeads (Miltenyi Biotec, #cat. 130-092-073) to remove memory T cells from the CD8+ T cell fraction, or (ii) the Human Naïve CD8 T Cell Isolation Kit (Miltenyi Biotec, #cat. 130-093-244). On day 0, naïve CD8 T cells were cocultured with peptide-pulsed (each peptide individually or by pool), unpulsed, or electroporated-mature moDCs in complete X-vivo 15 medium supplemented with IL-21 (30 ng/mL, Peprotech, Cranbury, NJ, USA, #cat. 200-21). On day 3, half the culture media was replaced with fresh culture media supplemented with 10 ng/mL IL-7 and IL-15 (final concentration: 5 ng/mL each) (Peprotech, #cat. 200-07 and 200-15 respectively). Cultures were observed periodically between days 5 and 10 for changes in medium color and cell confluence. Generally, cultures were split once or twice between days 5 and 10, and fresh culture media supplemented with IL-7 (final concentration: 5 ng/mL), IL-15 (final concentration: 5 ng/mL), and IL-2 (150 IU/mL, Peprotech, #cat. 200-02) was added. On day 10, freshly matured peptide-pulsed, unpulsed, or electroporated moDCs from the same donor were added to the cocultures. After 21 days of coculture, CD8 T cells were analyzed by tetramer staining.

#### 2.6.2. Tetramer Staining

According to the manufacturer’s instructions, peptide–HLA tetramers were produced using the UV-peptide exchange technology (Flex-T^TM^ Biolegend, San Diego, CA, USA). All manipulations were performed on ice. Equal volumes of synthetic peptide (400 µM) and peptide Flex-T monomer UVX (200 µg/mL) were mixed in a 96-well Polypropylene Microplate, V-shape (Greiner bio-one, Kremsmünster, Austria, cat.# 651201). The plate was centrifuged at 2500× *g* for 2 min at 4 °C before proceeding to peptide exchange under UV light (366 nm) for 30 min. Then, the plate was incubated in the dark for 30 min at 37 °C. For each peptide-exchanged monomer condition, half of the reaction volume was tetramerized using PE-streptavidin and the other half using APC-streptavidin. A total of 30 μL of peptide-exchanged monomer and 3.3 µL of PE- or APC-conjugated streptavidin were mixed and incubated on ice in the dark for 30 min. The reaction was then blocked using a 50 mM D-Biotin and 10% (*w*/*v*) NaN_3_ in PBS before incubating at 4 °C overnight or on ice for 30 min. 0.5 to 1 million CD8 T cells were stained for 30 min at 4 °C (using a mix of PE- and APC-conjugated tetramers). Stained cells were then analyzed with a FACS Celesta cytometer (BD Biosciences, Franklin Lakes, NJ, USA). For each peptide, proportions of double tetramer-positive cells were determined in CD8 T cells cocultured with unpulsed moDCs and CD8 T cells cocultured with peptide-pulsed moDCs (3 to 6 donors depending on the module) and electroporated moDCs (1 or 2 donors) (See Table S3 for details). The expansion fold of tetramer-positive CD8 T cells was calculated for each condition relative to the frequency observed in the unpulsed condition (naïve CD8 T cells cocultured with unpulsed moDCs). In cases where no tetramer-positive cell was detected in the unpulsed condition, the frequency was set to 1 tetramer-positive cell for this condition (Section 3.4). Results for frequencies of tetramer-positive cells and expansion folds are presented in Table S5.

#### 2.6.3. Functional Expansion of Antigen-Specific T Cells Followed by TCR Vβ CDR3 Sequencing (FEST Assay)

For the FEST assay experiment, coculture of naïve CD8 T cells and moDCs (pulsed with individual peptides or unpulsed) was performed using the same protocol as for tetramer staining experiments. On day 21, cells were harvested to perform TCR Vβ CDR3 sequencing. Uncultured naïve CD8 T cells from the same healthy donor were also used as another control condition. DNA was extracted from CD8 T cell samples using a QIAamp DNA blood mini kit (QIAGEN, #cat. 51104). TCR Vβ CDR3 sequencing was performed using the survey resolution of the immunoSEQ platform (Adaptive Biotechnologies, Seattle, WA, USA). Raw data exported from the immunoSEQ portal were processed with the FEST analysis web tool (www.stat-apps.onc.jhmi.edu/FEST, accessed on 15 December 2023) [38] with the following parameters: FDR 5%, Odds ratio 5, and minimum templates 5.

#### 2.6.4. PRIME 2.0 Score for Prediction of T-Cell Recognition

We used the PRIME2.0 web tool (http://ec2-18-188-210-66.us-east-2.compute.amazonaws.com:3000, accessed on 28 February 2023) [39] to predict T-cell recognition of our 48 TSAs. As controls, we used 14 immunogenic peptides extracted from the literature and 96 of the peptides used to train PRIME2.0 (Table S6).

## 3. Results

### 3.1. Selection of the Best TSA Candidates

The 91 TSAs we identified in EOC [24] are presented by 21 HLA allotypes (Figure S1a), with 1 to 15 TSAs per allotype. We focused on TSAs presented by the HLA-A or -B allotypes since HLA-C molecules are expressed at lower levels at the cell surface [40]. We prioritized HLA allotypes that presented numerous TSAs. Next, TSAs were selected based on (i) their HLA binding affinity and (ii) their expression in the TCGA ovarian cancer dataset (Figure S1b). In total, we selected 48 TSAs that are predicted binders for one of nine HLA allotypes: -A*01:01, -A*02:01, -A*03:01, -A*11:01, -A*29:02, -B*07:02, -B*08:01, -B*15:01, and -B*44:03. To facilitate their evaluation, these 48 TSAs were divided into 11 vaccine modules according to their HLA allotype specificity (Table 1 and Table S1, Figure 1a). Each module contained 2-5 peptides, and we assembled 1 or 2 modules per HLA allotype. The selected TSAs are predicted binders to HLA allotypes that are highly prevalent in several ethnicities (Figure S1c and Table S2; based on HLA frequency data from [30]). We also evaluated the frequency of expression of their RNA sequences in the TCGA ovarian cancer cohort using the proteogenomic tool BamQuery [31]. We found that all TSAs included in the present study are coded by transcripts expressed in 15 to 100% of the TCGA ovarian cohort, with 33 (69%) shared by at least 40% of the cohort (Figure 1b,c). Thus, the 48 selected TSAs are presented by highly prevalent HLA allotypes and shared by a sizeable fraction of EOC samples.

### 3.2. TSAs Are Well Presented by moDCs after Peptide Pulsing

**Figure 2:**
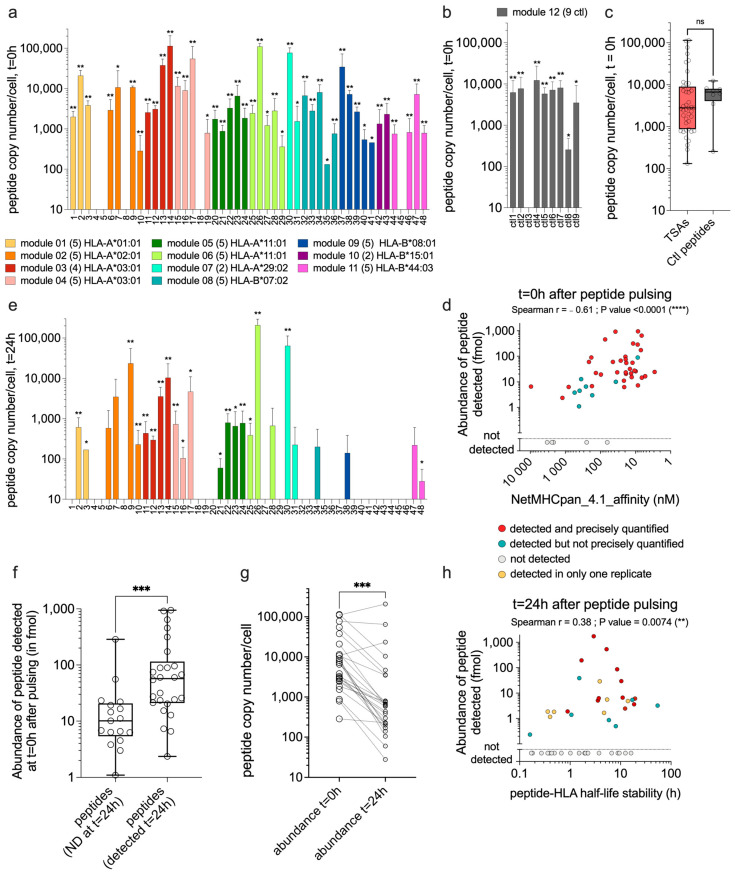
TSAs are well presented by moDCs after peptide pulsing. (**a**) Bars show the peptide copy number per cell detected by MS analyses for the 48 TSAs at t = 0 h after moDCs pulsing with individual synthetic peptide modules. Analyses were performed in three biological replicates (three different donors) (see Tables S1 and S3 for details on modules and donors, respectively). Each module is color-coded and annotated with its respective HLA and the number of TSAs. (**b**) Bars depict the peptide copy number per cell detected by MS analyses for nine control peptides known as immunogenic, at t = 0 h after moDCs pulsing with synthetic peptides (module 12; Table S4). The positive control peptides were analyzed for every donor included in these MS experiments, expressing the proper HLA (eight donors, three to four replicates for each HLA allotype of interest; see Table S3 for details on donors). (**c**) Peptide copy numbers obtained at t = 0 h after pulsing were compared between TSAs and positive control peptides using a non-parametric *t*-test (Mann–Whitney), showing no significant difference (ns) between these two groups. Each symbol represents the mean of replicates per peptide. (**d**) Correlation between the TSAs abundance detected by MS (in fmol) at t = 0 h after pulsing and the predicted binding affinity obtained from NetMHCpan_4.1 (low value in nM means high affinity). **** *p* < 0.0001. (**e**) The peptide copy number per cell was obtained from MS analysis for the 48 TSAs at t = 24 h after moDCs pulsing with peptide modules. Panels (**a**,**b**,**e**): ** detected and precisely quantified in at least two replicates, * detected but not precisely quantified in at least two replicates, no star = detected in only one replicate. (**f**) The abundance of peptides detected by MS at t = 0 h after pulsing (in fmol) for peptides detected (at least one replicate) or not by MS 24 h after pulsing. The two groups were compared using non-parametric *t*-tests (Mann–Whitney), ***; *p*-value = 0.0002. (**g**) Comparison of peptide copy number/cell detected by MS at t = 0 h and t = 24 h after peptide pulsing for each peptide detected in at least one replicate, 24 h after peptide pulsing. The two groups were compared using a Wilcoxon matched-pairs signed rank test, ***; *p*-value = 0.0005. (**h**) Correlation between the TSAs abundance detected by MS (in fmol) at t = 24 h and the predicted half-life stability of peptide–HLA complexes obtained from NetMHCstabpan_1.0. **; *p*-value = 0.0074. Panels (**d**,**h**): Spearman correlation coefficients and *p* values are indicated below the titles.

It is generally accepted that initiating a robust immune response requires antigen presentation by dendritic cells [41]. Therefore, we used sensitive targeted MS analyses to quantify the presentation of our 48 TSAs at the cell surface of moDCs after synthetic peptide pulsing. Human moDCs were pulsed for two hours with each module individually (2 to 5 TSAs per module; see Figure 1a and Table S1 for module details). Then, the presentation of TSAs at the moDC cell surface was assessed by MS at two different time points to evaluate the abundance and duration of TSA presentation (t = 0 h and t = 24 h after pulsing). Each TSA module was tested in three biological replicates (three different donors expressing the HLA of interest; see Table S3 for donor details). At t = 0 h, a control module (module 12) was also tested for all donors included in the study (eight donors in total, Table S3). This module contains nine positive control peptides known as immunogenic (one peptide per HLA of interest) and mostly of viral origin (Table S4). At t = 0 h, we found comparable proportions of peptides detected between TSAs and control modules. Indeed, 90% of the TSAs (43/48) and 89% of the control peptides (8/9) were detected by MS in at least two replicates (Figure 2a and Figure 2b, respectively). Moreover, the mean peptide copy numbers were similar for TSAs and control peptides (Figure 2c). We conclude that HLA class I allotypes present TSAs and immunogenic viral peptides with comparable efficiency.

Most but not all MHC-associated peptides have strong MHC binding affinity [42]. Accordingly, though TSAs used in this study are predicted to bind HLA allotypes with a good affinity, the majority are considered strong binders by the NetMHCpan4.1 prediction algorithm [28] (Figure S2a). Per our study design, all peptides in a module bind the same HLA allotype and compete for HLA binding. We observed a strong correlation (Spearman *p* < 0.0001) between the predicted binding affinity of a TSA for its cognate HLA allotype and its MS-estimated abundance MS at t = 0 h (Figure 2d). MS did not detect six peptides at t = 0 h (five TSAs and one control peptide). The lack of detection of four of these can be attributed to their relatively low MHC binding affinity compared to other TSAs in the same module: p4, p5 from module 01, p18 from module 04, and p45 from module 11 (refer to NetMHCpan_4.1_affinity in Table S1). The non-detection of peptides p8 and ctl3 could be related to lower stability in solution, high hydrophilicity, and/or higher limits of detection by MS.

The abundance of TSAs at the cell surface of moDCs was also evaluated 24 h after peptide pulsing. We found that 60% of the TSAs detected at t = 0 h were still detected 24 h after peptide pulsing in at least one replicate (26/43) (Figure 2e). Peptide abundance at t = 0 h positively correlated with detection at t = 24 h (Figure 2f). Unsurprisingly, the number of peptide copies per cell for the 26 peptides detected at t = 24 h was significantly lower than their abundance detected at t = 0 h (Figure 2g). These results are consistent with the half-life stability of these TSA–HLA complexes predicted by the NetMHCstabpan1.0 database [29] (Figure 2h). The predicted half-life stability of these peptide–HLA complexes positively correlated with peptide abundance detected by MS 24 h after pulsing (Figure 2h). These results suggest that, in addition to the HLA binding affinity, the predicted half-life stability of peptide–HLA complexes contributes to the abundance of peptides at the cell surface, especially for maintaining TSA presentation over time. As expected, we found a strong correlation between the predicted binding affinity of the peptides for their HLA molecule and the half-life stability of these peptide–HLA complexes (Figure S2b). These results support the idea that predicted binding affinity and stability should be considered as an initial step to prioritize the best targets for cancer vaccine development.

### 3.3. TCR-Vβ CDR3 Sequencing after Functional Expansion

**Figure 3:**
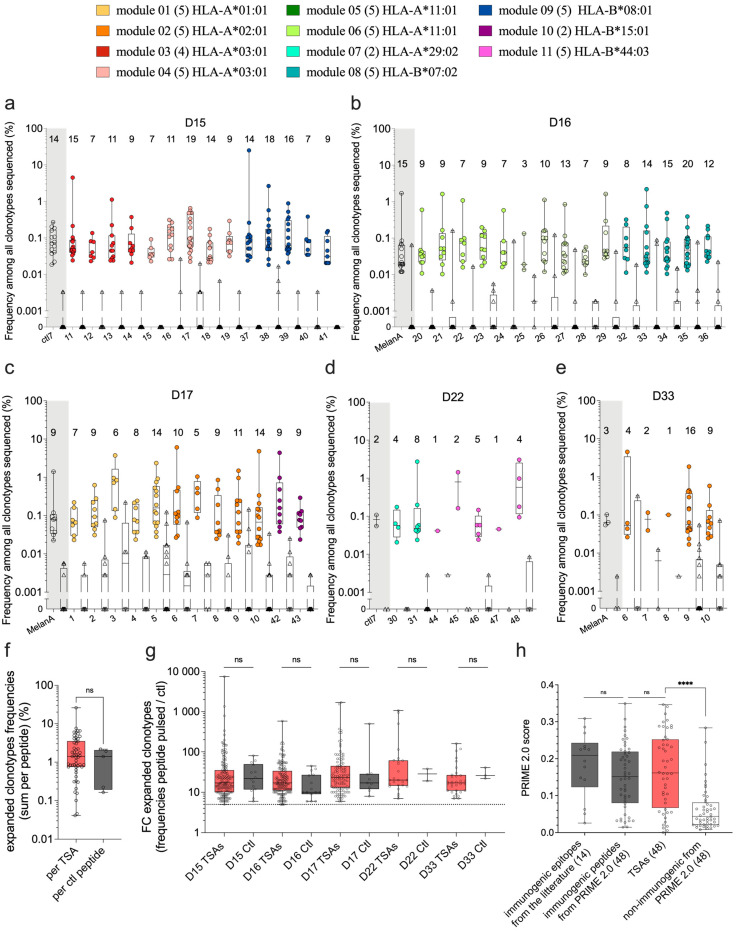
TCR Vβ CDR3 sequencing shows clonotype expansion following coculture with TSA-pulsed moDCs. Naïve CD8 T cells were cocultured with moDCs pulsed with one peptide and compared to naïve CD8 T cells cocultured with unpulsed moDCs. TCR Vβ CDR3 sequences were analyzed using the web platform MANAFEST [38]. Only clonotypes classified as significantly expanded in one peptide-pulsed condition (compared to the unpulsed condition) are represented. (**a**) Frequencies of expanded clonotypes among all clonotypes sequenced in each sample obtained with donors D15, (**b**) D16, (**c**) D17, (**d**) D22, and (**e**) D33. Each symbol represents the frequency for one expanded clonotype in the peptide-pulsed condition (circles) and its respective frequency in the unpulsed condition (triangles). (**a**–**e**) As positive controls, known immunogenic peptides were included according to the HLA genotype of each donor (MelanA binding HLA-A*02:01 or ctl7 binding HLA-B*08:01, highlighted in grey). Each TSA was tested in one donor (panels (**a**–**d**)), except peptides from module 2 (HLA-A*02:01, TSAs 6 to 10 in orange) tested in 2 different donors (panels (**c**,**e**)). The number of significantly expanded clonotypes found per TSA or control peptide is represented above each sample. (**f**) The sum of all expanded clonotype frequencies was calculated for each peptide condition (Table S5). Each symbol represents one peptide condition, and results obtained from TSAs were compared to positive control peptides. (**g**) Fold change of expansion (frequency in [peptide-pulsed/unpulsed] condition) is represented for each significantly expanded clonotype (each point). Results are grouped by donors and compared between TSAs and positive control conditions. (**f**,**g**) pink boxes represent TSAs; grey boxes represent positive control peptides. (**h**) PRIME score prediction of T-cell recognition (Table S6): immunogenic control peptides extracted from the literature (14), the 48 TSAs analyzed in this study, and a selection of immunogenic and non-immunogenic peptides used to train PRIME2.0 (48 peptides each). Pink represents the TSAs, grey represents the groups of immunogenic peptides, and white represents the non-immunogenic peptides. (**f**–**h**) statistical analyses were done using non-parametric *t*-tests (Mann–Whitney), where ns stands for not significant; **** *p*-value < 0.0001.

The first method we used to study the immunogenicity of TSAs presented by moDCs was TCR Vβ CDR3 sequencing. Naïve CD8 T cells were cocultured with moDCs that were either unpulsed (negative control) or pulsed with individual TSAs. DNA was extracted to perform TCR Vβ CDR3 sequencing at the end of the coculture. Clonotype sequences were analyzed using the web platform MANAFEST, developed by Danilova and colleagues [38]. Clonotypes with significantly higher frequencies in TSA-pulsed relative to the unpulsed condition were considered to represent expanded TSA-specific CD8 T-cell clonotypes. The CD8 T-cell expansion against individual TSAs was analyzed in one donor per TSA (Figure 3a–e), except for the five peptides from module 2 (HLA-A*02:01), which were tested in two different donors (Table S3, Figure 3c,e in orange).

Two peptides with demonstrated immunogenicity were used as positive controls. Thus, MelanA (ELAGIGILTV, HLA-A*02:01) or ctl7 (ELRSRYWAI, HLA-B*08:01) was added in each experiment according to the HLA alleles of the donor (Figure 3a–e, grey parts and Table S4). We found significantly expanded TCR clonotypes for the 48 TSAs and the two positive control peptides tested (Figure 3a–e). The number of significantly expanded clonotypes in response to individual TSAs ranged from 1 to 20, and from 2 to 15 clonotypes in response to the positive controls (Figure 3a–e, see numbers above symbols). No significant difference was found between the frequencies of expanded clonotypes specific for TSAs vs. positive controls (Figure 3f and Table S5). Likewise, the fold-expansion of clonotypes specific for TSAs and positive controls were similar (Figure 3g). To further validate these results, we used the PRIME2.0 algorithm [39] to assess the TCR recognition propensity of our TSAs. We compared the PRIME2.0 score of our TSAs to the score of three peptide groups: (i) 14 well-documented immunogenic peptides, (ii) 48 (randomly selected) immunogenic neo-epitopes used to train PRIME2.0, and (iii) 48 (randomly selected) non-immunogenic neo-epitopes used to train PRIME2.0. The PRIME 2.0 score of our TSAs was similar to that of the positive controls and significantly higher than that of non-immunogenic peptides (Figure 3h and Table S6). We conclude from these experimental data and bioinformatic analyses that our TSAs are as immunogenic as viral and neo-epitopes from the literature.

### 3.4. Tetramer Staining after Functional Expansion

**Figure 4:**
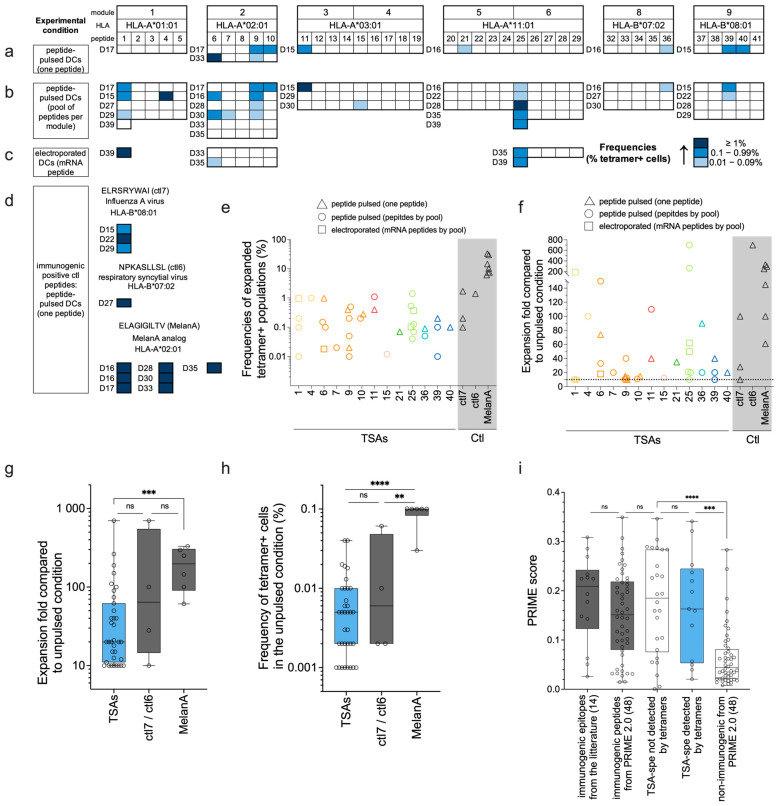
Expansion of tetramer-positive cells following coculture with TSA-loaded moDCs. Naïve CD8 T cells were cocultured with moDCs using different peptide loading experimental conditions and methods: (**a**) moDCs pulsed with each peptide individually, (**b**) moDCs pulsed with peptides pooled per module, and (**c**) moDCs electroporated with mRNA minigenes coding for peptides from modules 1, 2, or 6. Each peptide was tested in 3 to 6 different donors (donor codes are indicated on the left of each module; see details in Table S3) and with 2 or 3 different experimental conditions for peptide loading (**a**–**c**). The unpulsed moDCs condition is used as a negative control for specific expansion. (**d**) The positive control condition assessed the proportion of tetramer-positive cells following stimulation with moDCs pulsed with ctl6, ctl7, or MelanA (according to the donor HLA genotype) in each donor from panels (**a**–**c**). Panels (**a**–**d**): The frequencies of tetramer-positive CD8 T cells (in %) are represented following a blue intensity gradient. Tetramer-positive CD8 T cells were only considered expanded and described in the figure if expansion folds ≥ 10 compared to the negative control condition. (**e**) Frequencies of tetramer-positive CD8 T cells detected after functional expansion for TSAs and positive control peptides. (**f**) Compared to the unpulsed condition, the expansion fold of tetramer-positive CD8 T cells is represented for each TSA and positive control peptide. (**g**) The expansion fold of tetramer-positive CD8 T cells for all TSAs (in blue) was compared to the ctl7/ctl6 and MelanA groups. Individual points represent replicates of significantly expanded ag-specific T cells. (**h**) Frequencies of tetramer-positive CD8 T cells obtained in the unpulsed condition (see Section 2.6.2 for details). Panel (**e**–**h**): Only peptides for which expansion was observed in panels (**a**–**c**) or (**d**) are represented. (**i**) PRIME score comparison between TSAs groups for which we detected (13) or not (26) T-cell expansion by tetramers, immunogenic epitopes from the literature (14), and a selection of immunogenic or non-immunogenic peptides used to train PRIME2.0 (48 peptides each) (Table S6). Panels (**g**–**i**); groups were compared using non-parametric *t*-tests (Mann–Whitney), where ns stands for not significant; **** *p*-value <0.0001, *** *p*-value = 0.0009 (panel (**g**)) or *p*-value = 0.006 (panel **i**), and ** *p*-value = 0.0095 (panel (**h**)).

TCR Vβ CDR3-sequencing assays demonstrate the presence of TSA-responsive T cells in the naïve CD8 T cell repertoire but do not allow us to estimate their TCR avidity [43,44]. Since tetramer-positive cells present the highest TCR avidity [44], we performed p-HLA fluorescent tetramer staining of T cells primed with our 39 TSAs presented by HLA-A*01:01, -A*02:01, -A*03:01, -A*11:01, -B*07:02, and -B*08:01 molecules (all modules except module 7, 10, and 11; see Table S3). Modules 7, 10, and 11 were left out because their HLA (-A*29:02, -B*15:01, and -B*44:03) were not covered with the technology we used to perform these experiments. Naïve CD8 T cells were cocultured with moDCs loaded with TSAs using different experimental conditions (Figure 4a–c): moDCs pulsed with individual peptides or with a pool of peptides (per module) or moDCs electroporated with mRNA coding for TSA modules. mRNA electroporation was tested here as another technique for peptide loading, allowing endogenous processing and presentation for several TSAs (modules 1, 2, and 6). Indeed, using mRNA-based vaccines, peptides are produced and processed endogenously within the dendritic cells, which could result in more efficient loading and prolonged presentation.

As a control in each experiment, naïve CD8 T cells from each donor were cocultured with unpulsed moDCs (negative control) or moDCs pulsed with one peptide known to be immunogenic (positive control). Depending on the HLA restriction of the donor, the positive control peptide used in each experiment was either of viral origin (ELRSRYWAI/HLA-B*08:01; [38]) and (NPKASLLSL/HLA-B*07:02; [45]) named respectively ctl7 and ctl6 in Figure 2b and Figure 3a,d, or the modified epitope MelanA (ELAGIGILTV/HLA-A*02:01; [46]), (Tables S3 and S4 for details). The proportion of tetramer-positive cells was evaluated individually for each TSA or positive control peptide and compared to the unpulsed condition. Samples showing tetramer-positive frequencies with a 10-fold expansion compared to the unpulsed condition were considered positives and represented in blue in Figure 4a–d.

As expected, coculture with moDCs pulsed with the positive control peptides led to the detection of tetramer-positive T cells in all experiments (Figure 4d). We detected tetramer-positive CD8 T cells against 13/39 of the TSAs tested (33%) in at least one donor and/or peptide pulsing condition, with frequencies ranging from 0.01% to 1.4% (Figure 4a–c,e and Table S5). The TSA-specific populations were expanded 10 to 700 times compared to the unpulsed condition (Figure 4f and Table S5), comparable to the fold expansion obtained with the positive control peptides ctl6 and ctl7. Indeed, the ctl7-specific population shows frequencies ranging from 0.1 to 1.7%, while the frequency in the ctl6-specific population is 1.4% (one replicate) (Figure 4e). These results were comparable to those described in the literature for tetramer-positive ctl6-specific CD8 T cell frequencies after in vitro expansion [47]. For the MelanA-specific population, we showed frequencies of 6 to 33% of CD8 T cells after in vitro expansion (Figure 4e). This was unsurprising because this in vitro-created epitope is at the summit of the immunogenicity hierarchy [46,48,49,50].

Frequencies of tetramer-positive T cells highly varied between donors and even between experimental conditions from the same donor (Figure 4a–c,e, and Table S5), which could be explained by the low frequencies of antigen-specific cells in the naïve repertoire. This is the case for most of the peptide-specific T cells described in the literature, recognizing antigens of either viral or cancer origin, especially in the naïve compartment [48,49,51,52,53,54,55]. Indeed, we show that the frequencies of CD8 T cells specific to our TSAs and the positive control peptides (ctl6 and ctl7) in the unpulsed condition are within the same range (Figure 4h). These low precursor frequencies for antigen-specific T cells would introduce stochasticity in the expansion and detection of the tetramer-positive cells. In contrast, we observed that the frequency of MelanA-specific CD8 T cells is significantly higher in the unpulsed condition, compared to that of the TSAs and other positive controls (Figure 4h), leading to a higher expansion (Figure 4g) and frequency of MelanA-specific CD8 T cells (Figure 4e). This aligns with our and others’ results, showing that in vitro priming and detection of expanded specific T-cells is stochastic and usually observed in a fraction of the tested donors [55,56,57]. The data presented in this article highlights that the accuracy of TSA immunogenicity evaluation greatly benefits from an increased number of donors. Consistent with this assumption, the PRIME2.0 score was similar for TSAs that induced CD8 T-cell expansion detected by tetramers (suggesting higher avidity) or not (Figure 4i). This indicates that available prediction algorithms [39,58] and experimental data do not entirely overlap but complement each other.

In light of these experiments, five TSAs stand out: p1 (-A*01:01), p6 and p9 (-A*02:01), p25 (-A*11:01), and p39 (-B*08:01). We found CD8 T cell tetramer-positive cells against these TSAs in several different donors (Figure 4a–c). For three of these five TSAs (p1, p6, and p25), we observed a high expansion of ag-specific T cells in at least one replicate (expansion fold ≥ 150) (Figure 4f). Interestingly, we also found that these three TSAs were predicted to bind their cognate HLA molecules with a stronger affinity (lower nM value) than those other TSAs from their respective modules (see NetMHCpan_4.1_affinity (nM) column in Table S1). Three of the five TSAs (p6, p9, and p25) were detected by MS as presented by moDCs 24 h after peptide pulsing (Figure 2e), and two of these (p6 and p25) show the most prolonged predicted half-life stability for their cognate HLA compared to the other TSAs from their respective modules (see NetMHCstabpan column in Table S1). Moreover, these five peptides were coded by transcripts found in more than 40% of the TCGA ovarian cohort (Figure 1b). Considering their high immunogenic potential and high level of sharing between cancer samples, these five TSAs appear particularly interesting for TSA-based immunotherapy and should be prioritized.

## 4. Discussion

The strength of the initial T-cell response is influenced by the level and duration of presentation of the relevant peptide–HLA complexes in secondary lymphoid organs [59,60,61,62]. Thus, in the context of cancer vaccines, the capacity of TSAs to be well-presented by dendritic cells will also significantly influence the magnitude of T-cell responses that will emerge from the vaccination. We postulate that the careful quantification and optimization of TSA presentation by moDCs can improve the design of TSA vaccines. We focused on quantifying TSAs presented at the surface of moDCs using targeted MS analyses. We have shown that most of the TSAs investigated (90%) can be detected by MS after peptide pulsing on moDCs and that the abundance of peptide presentation strongly correlated with the predicted binding affinity of these peptides for their cognate HLA molecules (Figure 2d). In this study, the TSAs were tested by modules, which could lead to a potential competition between TSAs from the same module for binding on their HLA allotypes. Although this effect was minimized by regrouping a maximum of five TSAs in each module, the presentation at the cell surface could have been decreased for some peptides due to this competition. Hence, pulsing with individual peptides might improve the presentation of those with lower MHC-binding affinity.

According to NetMHCPan4.1 binding predictions, 10/48 TSAs and the modified MelanA epitope (ELAGIGILTV) are weak binders for their respective HLA allotypes (percentile rank binding affinity above the threshold of strong binders of 0.5%, Figure S2a and Table S1). As we (Figure 4e,f) and others [48,63,64,65,66,67,68] have shown that ELAGIGILTV induces robust CD8 T-cell responses, we conclude that predicted weak binders can be highly immunogenic. Among the 10 TSAs qualified as weak binders by NetMHCPan4.1, 7 were detected by MS at t = 0 h after pulsing. In contrast, the three others were not detected (p4, p5, and p45), indicating that other parameters also influence the detection by MS. Interestingly, while we did not detect p4 (HLA-A*01:01) on moDCs after peptide pulsing, it induced a significant expansion of tetramer-positive CD8 T cells in one donor (D15), which was also used for MS experiments (Figure 2a and Figure 4b, Table S3). Extremely low abundances, as low as a single copy per cell, can induce a T-cell response [69]. Thus, the amount of p4 presented by moDCs from D15 was probably below the limit of detection by MS but sufficient to trigger a specific T-cell expansion.

Several parameters can influence peptide detection sensitivity by MS, including low stability of peptides in solution, hydrophobicity, ionization efficacy, or losses during the immunoprecipitation process [70,71]. Despite their prediction as strong HLA binders, p8 and p18 were not detected by MS at t = 0 h. We speculate that their detection by MS was decreased because of either one or several of the parameters mentioned above. One control peptide (ctl3) was also not detected by MS after peptide pulsing. This peptide’s high positive charge probably resulted in a limited capacity factor (k’) and irreproducible MS detection [72]. Overall, the high detection rate of TSAs (90% at t = 0 h) and their abundance comparable to immunogenic viral peptides after moDC pulsing evocate a good immunogenic potential. Since the predicted binding affinity of peptides for their cognate HLA strongly correlated with their presentation level after pulsing, it would be a sensible parameter for the prioritization of TSAs to be included in vaccines.

We also noticed that the binding competition for HLA molecules could sometimes reduce TSA abundance at the cell surface and restrain their MS detection. Multi-peptide vaccines are more effective than single-peptide formulations as they mitigate the risk of immune escape [73,74]. However, if multiple tumor-specific antigens are present in a vaccine formulation, it is also essential to consider the possibility of competition for presentation on the surface of dendritic cells. Indeed, Aurisicchio and colleagues have shown that some epitopes can suffer from competition with other epitopes, both when delivered in the format of a minigene or as a peptide mixture [75]. From a vaccine design point of view, it can be deleterious to include TSA candidates that will compete for HLA binding but will not induce a strong T-cell response. This means that optimal vaccine design should include the testing of individual peptides.

TSAs tested herein were initially identified by untargeted liquid chromatography-tandem MS (LC-MS/MS) [24]. In this article, we detected and quantified most of the TSAs by sensitive targeted LC-MS/MS after pulsing of moDCs. This method is the only one that can directly and quantitatively estimate the number of TSA peptide copies presented by moDCs. However, immunoprecipitation-based HLA peptide isolation cannot distinguish HLA-restricted peptides on the cell surface from intracellular HLA–peptide complexes, which is unsuitable for immunotherapy applications [76,77]. Still, we observed increased frequencies of TCR clonotypes with Vβ CDR3 sequencing after functional expansion protocol for all the TSAs tested, thus validating their surface presentation after individual pulsing on moDCs.

A key factor regulating TSA immunogenicity is the presence of TSA-specific CD8 T cells in the pre-immune repertoire. In the present study, we evaluated the immunogenicity of TSAs using two different techniques: TCR Vβ CDR3 sequencing and tetramer staining after functional expansion. TCR Vβ CDR3 sequencing showed that CD8 T cell clonotypes could expand against all the TSAs tested. Although this assay appears highly sensitive for detecting TSA-specific CD8 T cells, it gives no information about their structural and functional avidity. Moreover, tetramer staining is the gold standard tool for monitoring ag-specific T cells but has been shown to require a TCR-pMHC affinity exceeding what T-cell activation requires [78]. This affinity threshold means tetramer staining can fail to detect functional T cells [78,79,80,81]. Accordingly, we detected tetramer-positive CD8 T cells for 33% (13/39) of the TSAs, while sensitive TCR Vβ CDR3 sequencing results revealed expanded TCR clonotypes for all of them. Tetramers are particularly useful for detecting and isolating antigen-specific T cells but are not, per se, a measure of functionality. Thus, adding functional assays, such as ELISpots and cytotoxicity assays, would undoubtedly improve the prioritization of TSA targets. Another group has explored the immunogenicity of non-canonical HLA-I tumor antigens identified through proteogenomics and shown that at least some of them are immunogenic (IFNg production and upregulation of 4-1BB) and shared across patients [82]. Their and our data reinforce the potential of TSAs as targets for off-the-shelf vaccines or TCR-based immunotherapies [83]. Furthermore, a multi-target approach in immunotherapy can enhance treatment effectiveness, improve patient outcomes, and decrease the likelihood of cancer recurrence [84], positioning immunogenic TSAs as appealing targets for designing multi-epitope vaccines.

Predicted peptide–HLA (p-HLA) binding affinity and stability correlated with peptide presentation levels measured by targeted MS (Figure 2d,h). p-HLA stability was shown to influence immunogenicity [85], and our data reinforce the idea that p-HLA affinity and stability are essential factors to consider when prioritizing targets. However, while these parameters predict antigen presentation, they do not predict T-cell recognition. Indeed, p-HLA binding affinity and stability did not correlate with immunogenicity testing with tetramer staining or TCR Vβ CDR3-sequencing assays. Immunogenicity prediction is complex and challenging as many factors govern it. In addition, selecting “true” non-immunogenic peptides to train the algorithms is difficult. Indeed, a negative T-cell assay result only means that a peptide failed to induce a T-cell response in that particular experiment. A failed expansion could be explained by the stochasticity in any naïve CD8 T-cell sampling. Ag-specific T cell frequencies in PBMCs can be as low as one tetramer-positive cell per ten million CD8 T cells [55]. Furthermore, there are important interindividual differences in the TCR repertoire [86]. Hence, the limited sensitivity and scalability of in vitro immunogenicity assays commonly yield false-negative results, thereby creating noise in the datasets used to train immunogenicity prediction tools.

In this study, we used moDCs to evaluate TSA presentation and immunogenicity. In a few replicates, we tested mRNA electroporation in addition to peptide pulsing to induce endogenous processing and presentation of TSAs. Notably, electroporated and peptide-pulsed DCs led to similar expansion levels of tetramer-positive T cells (Figure 4a–c and Table S5). Nonetheless, the yield and quality of moDCs exhibit significant interindividual differences, and these dendritic cells are less effective at T-cell stimulation than genuine conventional DCs [87,88,89]. Given the limitations of moDC vaccines, mRNA vaccines present several attractive features for clinical usage. The remarkable progress and efficacy demonstrated by mRNA-based vaccines against SARS-CoV-2 have underscored the transformative potential of this technology. Beyond their short manufacturing process and excellent safety record, mRNA vaccines present diverse design options to enhance antigen translation or interferon signaling [90,91,92,93,94,95,96]. Thus, several clinical trials of mRNA-based cancer vaccines are ongoing [97]. In this context, our study reinforces the idea that a careful evaluation of two parameters is required to optimize the design of anti-tumor vaccines: (i) HLA binding competition among epitopes included in a TSA-encoding mRNA and (ii) the capacity of each epitope to induce a robust immune response.

## 5. Patents

Université de Montréal has filed patent applications covering ovarian-cancer-specific antigens whose immunogenicity was evaluated in the present study.

## 6. Conclusions

Mass spectrometry analyses enabled accurate quantitative evaluation of TSA presentation. When multiple TSAs are presented together, they may compete for occupancy of HLA molecules. TCR Vβ CDR3 sequencing showed that aberrantly expressed ovarian cancer TSAs are immunogenic for naïve CD8 T cells from healthy donors.

## Figures and Tables

**Table 1:** 48 TSAs peptides used in this study.

Peptide Sequence (This Study)	Module ID	Peptide ID
TSDRLFLGY	01	1
ESDEQTLNY	01	2
YLDTAQKNLY	01	3
TPSSHLGLLSY	01	4
LSGCCSLY	01	5
LLLDKLYFL	02	6
SLLNVIGLSV	02	7
SLIPTALSL	02	8
LLSSKLLLM	02	9
IIHSSSLLL	02	10
RTATPLTMKK	03	11
VLAGTVLFK	03	12
RTATPLTMK	03	13
SVYMATTLK	03	14
RLATAPSEK	04	15
RLVTEPSGPK	04	16
RMKTFMMSH	04	17
RLLLPLQSR	04	18
ATWQSVLAR	04	19
STQMTITTQK	05	20
TTLKYLWKK	05	21
GTSPPSVEK	05	22
GTAQVGITK	05	23
SSALLAVALK	05	24
ATLQAAILYEK	06	25
LVFNIILHR	06	26
ASNPVIKKK	06	27
SSSALLAVALK	06	28
RTHQMNTFQR	06	29
VYMATTLKY	07	30
TLVVSIIIY	07	31
SPQTQTHTL	08	32
RPGAGPPGIL	08	33
RVKSTISSL	08	34
PSPLRPSL	08	35
NPSEGSGIRL	08	36
YLATKFMPI	09	37
TPKLRETSV	09	38
YGLPRVVAV	09	39
DILGKSLTL	09	40
YLKWAQIL	09	41
IMKKIRESY	10	42
SQGFSHSQM	10	43
AEHQEGTGTW	11	44
TEISNSQAA	11	45
KEVDPASNTY	11	46
AEEEIMKKI	11	47
AETGVKKPQ	11	48

## Data Availability

The original contributions presented in the study are included in the article/Supplementary Material, further inquiries can be directed to the corresponding author.

## Supplementary Materials

The following supporting information can be downloaded at https://www.mdpi.com/article/10.3390/curroncol31060236/s1, Figure S1: Selection of nine high-frequency alleles presenting several TSAs; Figure S2: Predicted HLA binding and stability of the 48 TSAs; Table S1: List of TSA peptides used in the study; Table S2: The prevalence of HLA allotypes included in the study in four populations; Table S3: List of blood donors used in the study with their HLA allotypes; Table S4: List of control peptides used in the study; Table S5: Frequencies and expansion folds obtained from TCR Vβ CDR3 sequencing and tetramer experiments; Table S6: Results obtained from PRIME2.0 algorithm (Figure 3h and Figure 4h).
