# Supplementary material for: Immunogenicity of Non-Mutated Ovarian Cancer-Specific Antigens
Source: Curr Oncol. 2024 May 30;31(6):3099–121. doi: 10.3390/curroncol31060236 (PMC11203340; doi:10.3390/curroncol31060236)
Supplement: Supplementary file 1 [file curroncol-31-00236-s001.zip › Supplementary figures.pdf]

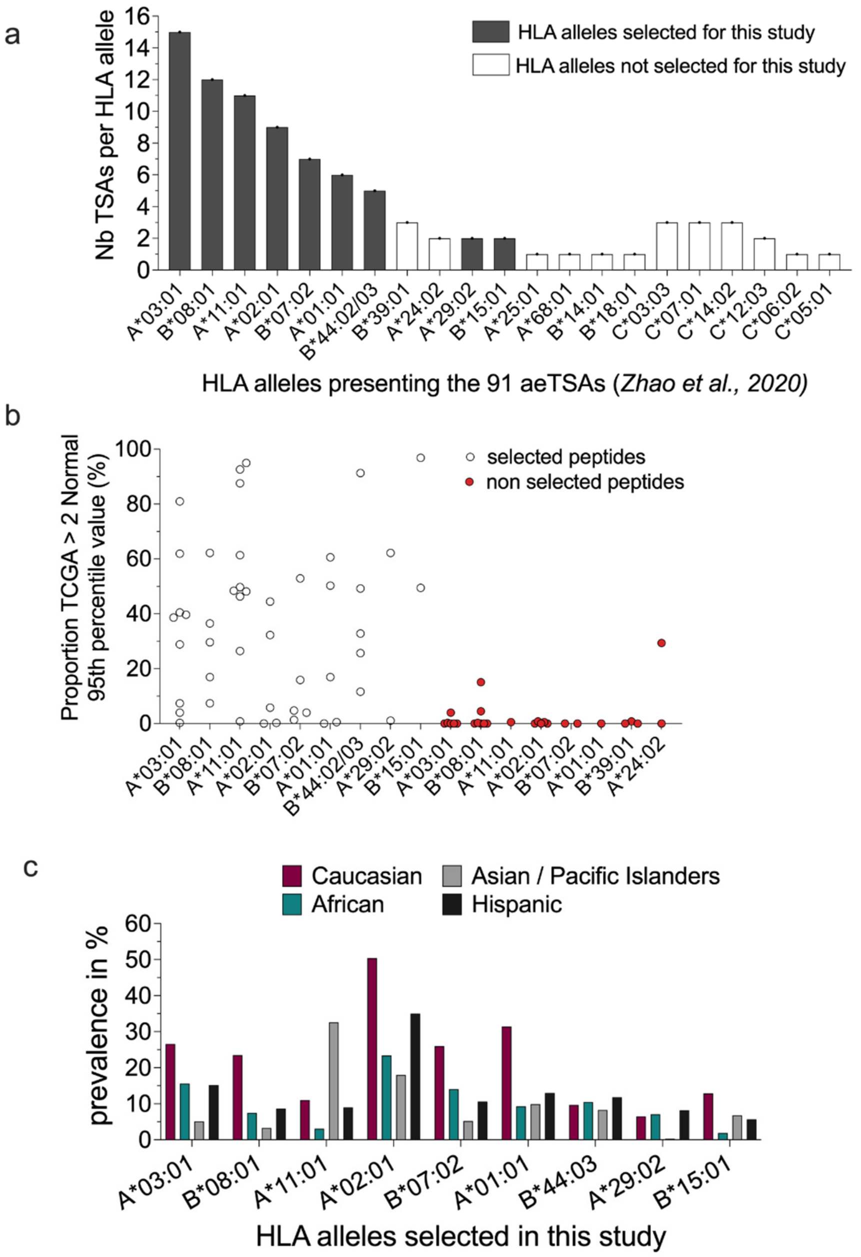

Figure S1. Selection of 9 highly expressed HLA alleles with several TSA candidates.

(**a**) Repartition of the 91 tumour-specific antigens (TSAs) published in our previous study (Zhao et al., 2020) according to their HLA allele. (**b**) The proportion of TCGA samples with a TSA-coding sequence expression higher than two times the normal 95th percentile value is represented for each TSA. The normal 95th percentile value corresponds to the 95th percentile expression value calculated from all normal tissue samples used in the study (mTECs (n = 11) + GTEx (except testis) (n = 2439)). (**c**) Prevalence of the nine HLA alleles selected for the present study in 4 different populations based on data from [30].

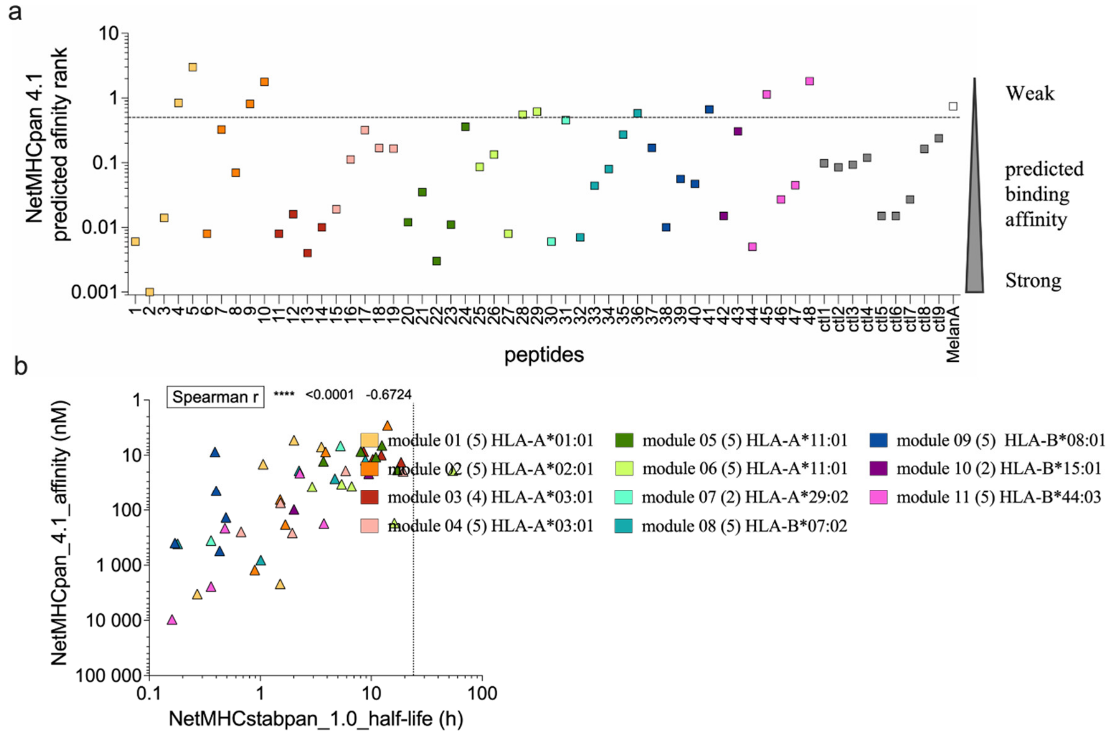

Figure S2: Predicted HLA binding and stability of the 48 TSAs.

(**a**) Predicted binding affinity rank of each peptide for the relevant HLA allele obtained from NetMHCpan4.1 database. The dotted line represents the threshold for predicted strong binders (0.5%). (**b**) Correlation between predicted binding affinity (in nM from NetMHCpan4.1; lower is the value, higher is the affinity) and predicted half-life stability for peptide-HLA complexes (in hours from NetMHCstabpan1.0) for the 48 tumour-specific antigens (TSAs). Legend for colours is represented (TSAs = peptides 1 to 48), and grey and white symbols represent control peptides (ctl1 to 9 and MelanA in panel (**a**)).
